# War Wastage

**Published:** 1918-09

**Authors:** 


### WAR WASTAGE.

" The Sources of Wastage of Men in War, with Suggestions for their Amelioration " was the subject under discussion at the second session of the Research Society. Lieutenant-General Burtchaell, Director-General of the Medical Service of the British armies in France, in opening the discussion gave the experience of the British armies, and graphically described the methods that have been employed to reduce the incidence of sickness among their troops, as well as those which have been found most effective in getting the wounded back into the line at the earliest possible moment. His remarks, as published in this number of *War Medicine* represent one of the most authoritative, as well as one of the most practical, expositions of the subject that have appeared in medical literature.

Colonel H. Ensor told of the " Wastage from Diseases Due to Lice " in which he showed that the lowly " crab " or " cootie ", in causing dermatitis, trench fever and typhus, is almost as much of an enemy to the human race generally, and to the Allied armies in particular, as the obligate anaerobic saprophytic parasite — the genus hominis Germani. Col. Ensor told of wastage due to lice in the British Army during the earlier years of the war, and of the de-lousing methods that had been employed, which have materially reduced the incidence of sickness among the British troops.

Lt.-Col. Brown, in discussing the " Prevention of Wastage in Convalescent Depots " gave the history and evolution of the convalescent camps of the British armies, and told of how the lightly wounded and the convalescent sick were built up and hardened both in body and morale to get back to the line as fighting men in the shortest period of time.

Major Cowie discussed the part played by the regimental surgeon in preventing wastage of men. He kept the audience in a roar with his inimitable wit and humor, but at the same time he impressed upon the medical officers present many important facts regarding the duties and opportunities of the surgeon at the Front in preventing the loss of men from the firing-line.

General Burtchaell here gave a humoros account of an incident that happened to Major Cowie during a battle. He said that a shell exploded under Major Cowie's horse, and that the regiment was horrified to see the horse and rider go up in the air. After a time Major Cowie came down and did not lose a minute in dressing the wounds of the soldiers, but the horse was never heard from again.

Médecin-Major Hautant read a paper on " The Most Frequently Observed Oto-Laryngeal Affections and Wounds, and the Means of Treatment to Avoid Wastage ". He recited the experience of the French army in dealing with oto-laryngeal cases, and showed the importance of the prevention of acute infections like tonsilitis and otitis, and spoke of how wounds of the ear are dealt with to prevent deafness and chronic middle ear disease.

